# Electrical stimulation drives chondrogenesis of mesenchymal stem cells in the absence of exogenous growth factors

**DOI:** 10.1038/srep39302

**Published:** 2016-12-22

**Authors:** Hyuck Joon Kwon, Gyu Seok Lee, Honggu Chun

**Affiliations:** 1 Department of Physical Therapy and Rehabilitation, College of Health Science, Eulji University, Gyeonggi, Republic of Korea; 2 Department of Microbiology and Molecular Biology, College of Bioscience and Biotechnology, Chungnam National University, Daejeon, Republic of Korea; 3 Department of Bio-convergence Engineering, Korea University, Seoul, Republic of Korea

## Abstract

Electrical stimulation (ES) is known to guide the development and regeneration of many tissues. However, although preclinical and clinical studies have demonstrated superior effects of ES on cartilage repair, the effects of ES on chondrogenesis remain elusive. Since mesenchyme stem cells (MSCs) have high therapeutic potential for cartilage regeneration, we investigated the actions of ES during chondrogenesis of MSCs. Herein, we demonstrate for the first time that ES enhances expression levels of chondrogenic markers, such as type II collagen, aggrecan, and Sox9, and decreases type I collagen levels, thereby inducing differentiation of MSCs into hyaline chondrogenic cells without the addition of exogenous growth factors. ES also induced MSC condensation and subsequent chondrogenesis by driving Ca^2+^/ATP oscillations, which are known to be essential for prechondrogenic condensation. In subsequent experiments, the effects of ES on ATP oscillations and chondrogenesis were dependent on extracellular ATP signaling via P2X_4_ receptors, and ES induced significant increases in TGF-β1 and BMP2 expression. However, the inhibition of TGF-β signaling blocked ES-driven condensation, whereas the inhibition of BMP signaling did not, indicating that TGF-β signaling but not BMP signaling mediates ES-driven condensation. These findings may contribute to the development of electrotherapeutic strategies for cartilage repair using MSCs.

Articular cartilage is a unique load-bearing tissue that lacks vascular, neural, and lymphatic tissues. Articular cartilage cannot spontaneously regenerate *in vivo* because chondral defects do not penetrate the subchondral bone and therefore cannot be accessed by blood supply or mesenchymal stem cells (MSCs) from bone marrow^1^,^2^. Hence, researchers and surgeons have developed various techniques to repair cartilage tissues^3^. However, most current cartilage repair techniques eventually lead to the formation of fibrocartilage and cartilage degeneration^4^. Accordingly, autologous chondrocyte implantation has been used for cartilage repair but is associated with several disadvantages, including limited cell sources and frequent injury of healthy cartilage during surgery, further encouraging formation of inferior fibrocartilage at defect sites^5^.

Owing to their capacity for self-renewal and differentiation into adipocytes, cartilage, bone, tendons, muscle, and skin, MSCs are an attractive cell source for cartilage defect therapies^6^,^7^,^8^,^9^,^10^,^11^. Furthermore, because MSCs are free of both ethical concerns and teratoma risks, MSCs have considerable therapeutic potential^12^. Therefore, it is important to develop effective and safe methods for the induction of MSC chondrogenesis and for the production of stable cartilaginous tissue by these cells. Multiple previous studies have demonstrated the effects of various chemical factors, such as soluble growth factors, chemokines, and morphogens, on chondrogenesis. In particular, transforming growth factors (TGF-β) and bone morphogenetic proteins (BMPs) have been shown to play essential roles in the induction of chondrogenesis^13^,^14^. Although these growth factors have great therapeutic potential for cartilage regeneration, growth factor-based therapies have several clinical complications, including high dose requirements, low half-life, protein instability, higher costs, and adverse effects^15^,^16^. Recent studies demonstrate that physical factors regulate cell differentiation and tissue development^17^,^18^,^19^,^20^,^21^, suggesting therapeutic potential of physical factors as alternatives to chemical agents for cartilage regeneration.

Endogenous electrical signals have been observed in articular cartilage during physiological processes, prompting the application of various electrical stimulation (ES) and electromagnetic field (EMF) inducers to *in vitro* chondrogenesis and *in vivo* cartilage repair^22^,^23^,^24^. In particular, studies using animal models show that ES and EMF improve healing of cartilage defects by increasing cell proliferation, glycosaminoglycan synthesis, and the expression of extracellular matrix genes, and by reducing the production of inflammatory mediators^25^,^26^,^27^,^28^,^29^. However, the precise roles of ES and EMF in cartilage repair remains unclear, and the effects of ES and EMF have only been observed in the surrounding cartilage, and not in articular defects. These observations indicate that ES or EMF alone have limited therapeutic efficacy for the repair of large osteochondral defects. Autologous chondrocyte transplants have been successfully used to repair large osteochondral defects^30^. Thus, multiple studies have investigated the effects of ES on proliferation and synthesis of cartilage extracellular matrix proteins in chondrocytes^31^,^32^,^33^,^34^. However, chondrocytes gradually decrease in number with age^33^, and it is difficult to obtain sufficient chondrocyte numbers to repair large defects due to limited life span and de-differentiation with downregulation of cartilage-marker genes during culture^34^,^35^. As an alternative, MSCs with self-renewing abilities can differentiate into chondrocytes and offer a reliable resource for ES-based therapies for damaged cartilage defects. However, although recent studies have reported the effects of ES on proliferation and differentiation of MSCs^36^,^37^, it remains unclear whether ES induces chondrogenic differentiation of MSCs.

Our previous studies demonstrated that intracellular ATP levels oscillate during chondrogenic differentiation and the ATP oscillations play critical roles in prechondrogenic condensation^38^,^39^, and that extracellular ATP signaling mediates the ATP oscillations during chondrogenesis^40^. ES has been shown to activate extracellular ATP signaling in a variety of cell types^41^,^42^,^43^. Therefore, we hypothesized that ES induces ATP oscillations in MSCs by stimulating extracellular ATP signaling and consequently drives MSC chondrogenesis. In the present study, we demonstrated that ES induces ATP oscillations and promotes MSC chondrogenesis in the absence of exogenous growth factors. Moreover, ES induced MSC chondrogenesis more effectively than treatments with chondrogenic medium (CM) supplemented with soluble growth factors. Accordingly, further experiments showed that extracellular ATP signaling via P2X_4_ receptors was responsible for ATP oscillations and mediated chondrogenesis following ES. In addition, ES-driven chondrogenesis depended on both TGF-β and BMP signaling pathways. However, TGF-β signaling, but not BMP signaling, was involved in ES-driven condensation. The present data suggest that ES has high potential as an MSC-based therapy for cartilage regeneration.

## Results

### ES induces calcium/ATP oscillations and MSC condensation

**Figure 1:**
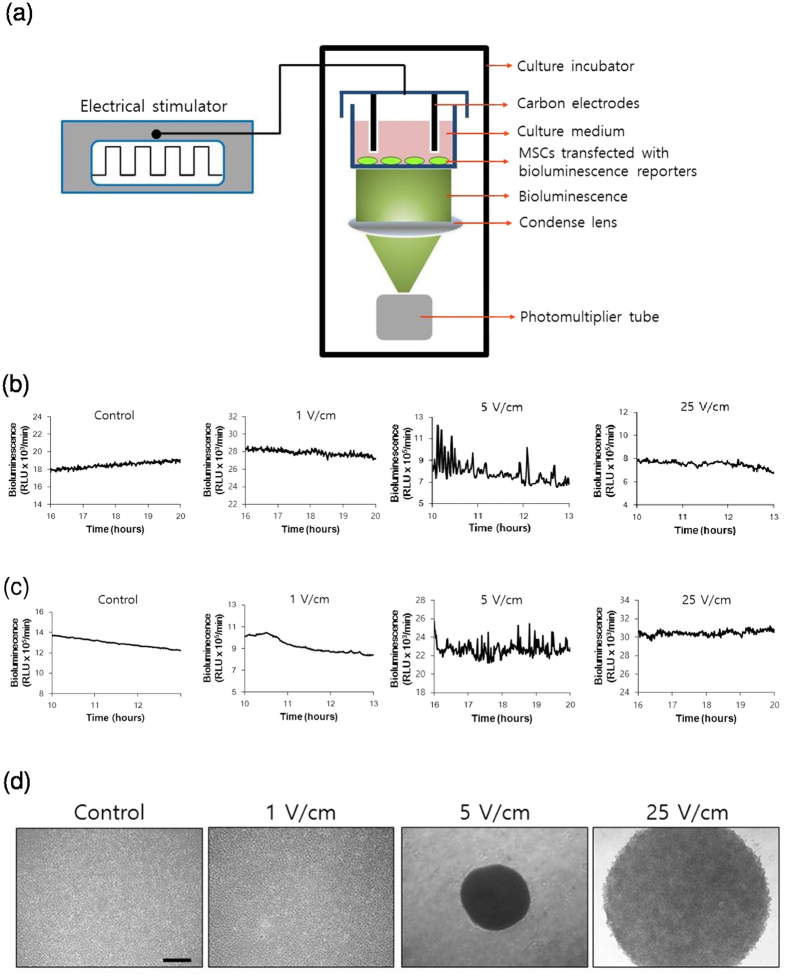
Electrical stimulation induces ATP/Ca^2+^ oscillations and MSC condensation. (**a**) Schematic of the real-time bioluminescence monitoring system for ES treated MSCs transfected with bioluminescence reporters (**b**) Real-time monitoring of intracellular ATP levels in MSCs under ES (0, 1, 5, and 25 V/cm, 8 ms, 5 Hz) using a bioluminescence ATP reporter (P_ACTIN_-Luc) (**c**) Real-time monitoring of intracellular Ca^2+^ levels under ES (0, 1, 5, and 25 V/cm, 8 ms, 5 Hz) using a bioluminescence Ca^2+^ reporter (P_CMV_-AQ) (**d**) Condensation behaviors of MSCs in micromass culture were examined using phase contrast images during culture for 3 days under ES (0, 1, 5, and 25 V/cm, 8 ms, 5 Hz); Scale bars, 500 μm.

Flow cytometry analysis showed that the expanded MSCs were positive for typical MSC markers (Sca-1, CD44, CD73) but showed low expression of markers of hematopoietic stem cells (CD34), macrophages (CD11b), and granulocytes (CD45), which confirmed that the expanded MSCs exibited the characteristics of MSCs (Supplementary Fig. S1). To determine whether ES induces ATP oscillations in MSCs, we monitored temporal changes in intracellular ATP levels using a bioluminescent ATP-dependent luciferase (Luc) reporter gene fused to a constitutive ACTIN promoter (P_ACTIN_-Luc). Following transfection of MSCs with P_ACTIN_-Luc, bioluminescence intensity was measured in real-time during ES of 0, 1, 5 or 25 V/cm at 5 Hz (Fig. 1a). In these experiments, ES of 5 V/cm induced ATP oscillations of ~ 5 min periods, whereas ES of 0, 1, or 25 V/cm did not (Fig. 1b). Because ATP oscillations were driven by changes in Ca^2+^ concentrations during chondrogenesis^38^, we examined Ca^2+^ oscillations using the bioluminescent Ca^2+^ reporter Aequorin (AQ) gene fused to a CMV promoter (P_CMV_-AQ)^44^. ES of 5 V/cm consistently induced Ca^2+^ oscillations, whereas ES of 0, 1, or 25 V/cm did not (Fig. 1c), suggesting that optimized ES can drive fluctuations of both Ca^2+^ and ATP. We previously showed that growth factors such as TGF-βs and insulin induce prechondrogenic condensation by generating Ca^2+^/ATP oscillations^39^. Consistently, ES of 5 V/cm which induced Ca^2+^/ATP oscillations led to compact condensation of MSCs, whereas ES of 0, 1, or 25 V/cm did not (Fig. 1d). Moreover, time-course observations showed that ES of 5 V/cm induced gradual aggregation of MSCs into compact structures within 3 days, corresponding with the effects of chondrogenic medium (CM) supplemented with growth factors such as TGF-βs and insulin (Supplementary Fig. S2). These results indicate that ES induces prechondrogenic condensation by driving Ca^2+^/ATP oscillations, even in the absence of exogenous growth factors.

### ES induces MSC chondrogenesis

**Figure 2:**
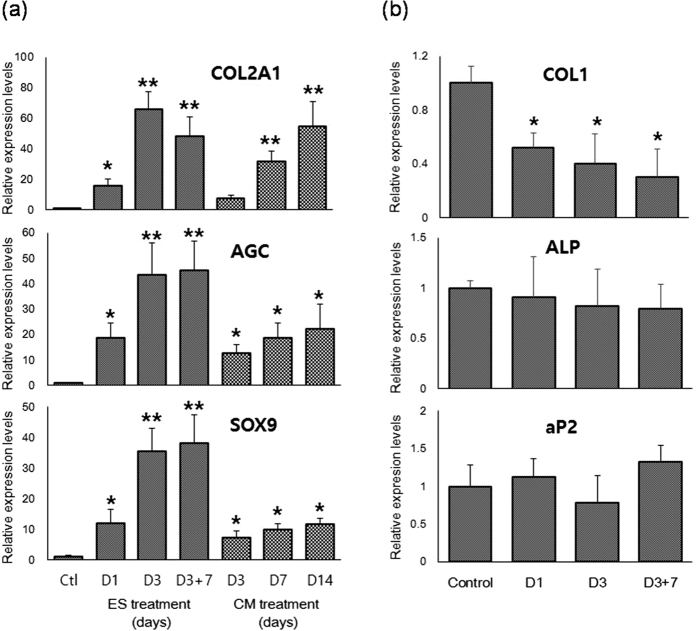
ES enhances mRNA expression of chondrogenic markers in MSCs. Relative levels of mRNA were determined by quantitative real-time PCR in relation to beta-actin. (**a**) Gene expression of type II collagen (COL2A1), aggrecan (AGC), and Sox9 in MSCs was determined at various time points during ES treatment (5 V/cm, 8 ms, 5 Hz) and CM treatment. Data are presented as means ± standard deviations (S.D.). Statistical analyses were performed using ANOVA (Dunnett’s test); **p < 0.01, *p < 0.05 versus Ctl (control; MSCs cultured in maintenance medium for 3 days). (**b**) Gene expression of type 1 collagen (COL1), alkaline phosphatase (ALP), and adipocyte protein (aP2) was determined at various time points during ES treatment (5 V/cm, 8 ms, 5 Hz). Data show mean ± S.D. Statistical analyses were performed using ANOVA (Dunnett’s test); **p < 0.01, *p < 0.05 versus control (MSCs cultured in maintenance medium for 3 days).

**Figure 3:**
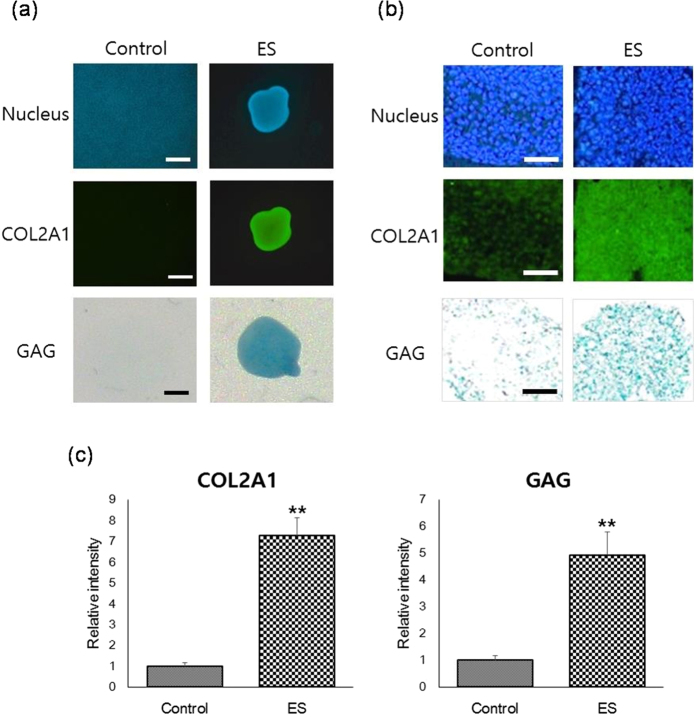
ES enhances expression of COL2A1 and GAGs in MSCs. (**a**) Immunofluorescent staining (COL2A1; green) and alcian blue staining (glycosaminoglycan (GAG); blue) of micromass cultures after culture in maintenance medium (control) or maintenance medium with ES (5 V/cm, 8 ms, 5 Hz) for 3 days (ES). Nuclei were stained blue using Hoechst 33342; Scale bars, 500 μm. (**b**) Immunofluorescent staining (COL2A1; green) and alcian blue staining (GAG; blue) of paraffin-embedded sections of micromass cultures after culture in maintenance medium (control) or maintenance medium with ES (5 V/cm, 8 ms, 5 Hz) for 3 days (ES). Nuclei were stained blue using Hoechst 33342; Scale bars, 100 μm. **(c)** Quantitative analysis of immunostaining intensity and alcian blue staining intensity. Data are presented as means ± S.D. and differences were identified using Students t-test, **p < 0.01.

In further experiments, the effects of optimized ES on chondrogenic differentiation were examined. Since ES for 3 days had little effect on cell damage (<5%) but induced significant cell death (almost 50%) after 7 days (Supplementary Fig. S3), ES was performed for 3 days. Gene expression of chondrogenic markers such as type II collagen (COL2A1), aggrecan (AGC), and SRY (Sex Determining Region Y)-Box 9 (SOX9)^45^,^46^,^47^ was analyzed at 1 and 3-day of ES treatment and 7-day post-ES treatment. Quantitative real-time RT-PCR analyses showed that ES significantly enhanced gene expression of chondrogenic markers within 3 days of ES treatment, revealing a 66-fold increase in COL2A1, a 43-fold increase in AGC, and a 35-fold increase in SOX9 expression at 3-day of ES (Fig. 2a). Moreover, increases of chondrogenic marker expression in MSCs treated with ES for 3 days were much higher than those in the CM exposed cells, and were greater than or equal to expression levels in MCSs that were fully differentiated into chondrocytes following treatment with CM for 14 days (Fig. 2a). These data suggest that ES induces MSC chondrogenesis more effectively than CM. Moreover, it was found that the chondrogenic markers were highly expressed for as long as 7 days after the last ES treatment (Fig. 2a), which confirmed that chondrogenesis was induced in MSCs by ES. In addition, ES led to significant decreases in the expression of type I collagen (COL1; Fig. 2b). This result indicates that ES induces differentiation of MSCs into not fibrocartilaginous tissues but hyaline cartilaginous tissues^48^,^49^,^50^,^51^. In contrast, ES did not significantly change the expression of the osteogenic marker alkaline phosphatase (ALP), or the adipogenic marker adipocyte protein 2 (aP2; Fig. 2b), indicating that ES does not induce osteogenesis or adipogenesis. Additionally, immunostaining and alcian blue staining analyses showed significantly higher expression of type II collagen and GAGs in ES-treated MSCs compared with control cells (Fig. 3a–c). Taken together, these data suggest that ES induces MSC chondrogenesis for hyaline cartilage regeneration even in the absence of exogenous growth factors.

### Extracellular ATP signaling via P2X_4_ receptor mediates ATP oscillations, condensation, and subsequent chondrogenesis following ES

**Figure 4:**
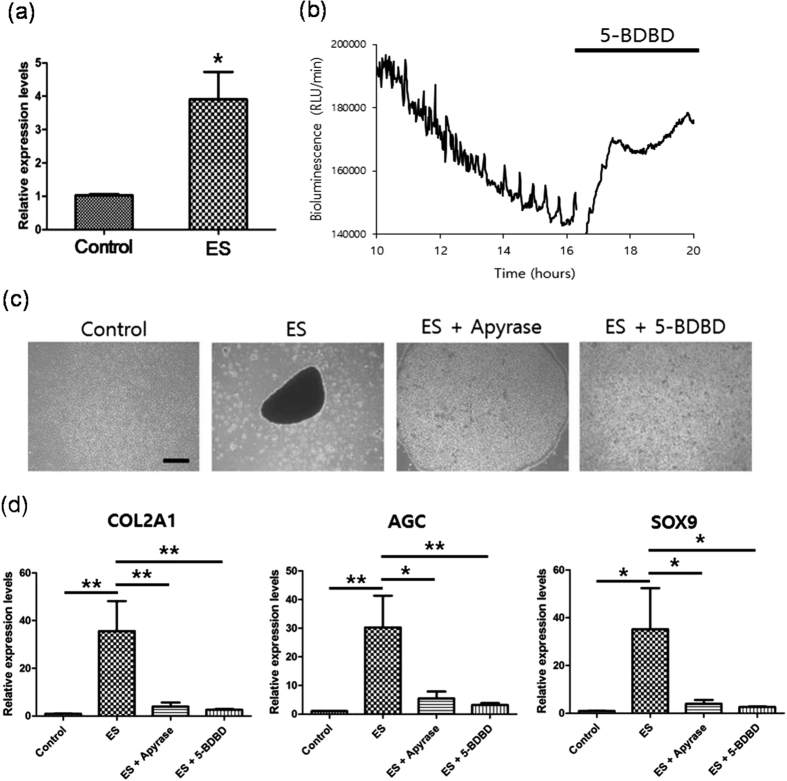
Extracellular ATP signaling via P2X_4_ receptors mediates ES-driven ATP oscillations, prechondrogenic condensation, and chondrogenesis. (**a**) Real-time gene expression analyses of P2X_4_ receptors in micromass cultures of MSCs after culture for 3 days in maintenance medium (control) or maintenance medium under ES. Data are presented as means ± S.D. and differences were identified using Students t-test, *p < 0.05 (**b**) Effects of 5-BDBD on ES-driven ATP oscillations; MSCs in micromass culture were treated with 5-BDBD after induction of P_ACTIN_-Luc oscillations by ES. Bioluminescence monitoring was performed after application of ES to MSCs (time = 0 h). (**c**) Effects of apyrase and 5-BDBD on ES-driven MSC condensation; MSCs in micromass culture were examined using phase contrast images after 3 days culture in maintenance medium (control), with ES (ES), with ES plus apyrase (ES + apyrase), or with ES plus 5-BDBD (ES + 5-BDBD); Scale bars, 500 μm. (**d**) Suppressive effects of apyrase and 5-BDBD on ES-induced type II collagen (COL2A1), aggrecan (AGC), and Sox9 expression. After 3 days culture in maintenance medium (control), with ES (ES), with ES plus apyrase (ES + apyrase), or with ES plus 5-BDBD (ES + 5-BDBD), gene expression was analyzed in MSCs using real-time PCR. Data are presented as means ± S.D. and differences were identified using ANOVA; **p < 0.01, *p < 0.05.

Extracellular ATP signaling via the P2X_4_ receptor reportedly plays key roles in prechondrogenic condensation by mediating ATP oscillations^36^. In the present study, ES enhanced the expression of P2X_4_ receptor mRNA in MSCs (Fig. 4a), suggesting that extracellular ATP signaling via the P2X_4_ receptor is involved in ATP oscillations, MSC condensation, and chondrogenesis following ES. In agreement, the P2X_4_ purinergic receptor inhibitor 5-BDBD inhibited ES-driven ATP oscillations (Fig. 4b). In subsequent experiments, it was examined whether extracellular ATP signaling via the P2X_4_ receptor was associated with ES-driven condensation and subsequent chondrogenesis. 5-BDBD almost completely inhibited ES-driven condensation, and apyrase significantly suppressed this process (Fig. 4c). In addition, ES did not enhance the expression of the chondrogenic markers COL2A1, AGC, and SOX9 in MSCs treated with either apyrase or 5-BDBD (Fig. 4d). Hence, extracellular ATP signaling via P2X_4_ receptors mediates ES-driven MSC condensation and chondrogenesis.

### Intercellular communications mediates ES-driven chondrogenesis

**Figure 5:**
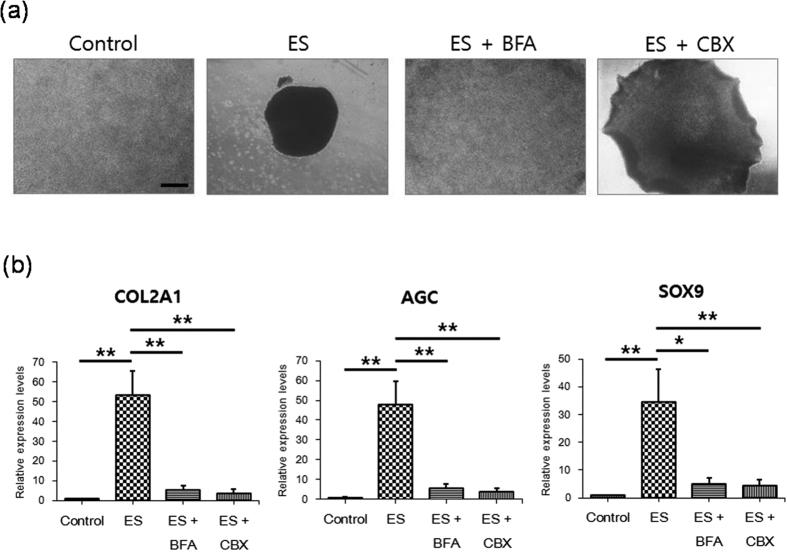
Paracrine signals and gap junction mediates MSC condensation and chondrogenesis following ES. (**a**) Effects of BFA and CBX on ES-driven MSC condensation; MSCs in micromass culture were examined using phase contrast images after 3 days culture in maintenance medium (control), with ES (ES), with ES plus BFA (ES + BFA), or with ES plus CBX (ES + CBX); Scale bars, 500 μm. (**b**) Suppressive effects of BFA and CBX on ES-induced type II collagen (COL2A1), aggrecan (AGC), and Sox9 expression. After 3 days culture in maintenance medium (control), with ES (ES), with ES plus BFA (ES + BFA), or with ES plus CBX (ES + CBX), gene expression was analyzed in MSCs using real-time PCR. Data are presented as means ± S.D. and differences were identified using ANOVA; **p < 0.01, *p < 0.05.

It was known that ES influences intercellular communications such as paracrine signaling^52^ and gap junction^53^. We found that BFA, which blocks classical secretion of paracrine factors, suppressed ES-driven condensation and ES-driven increases of COL2A1, AGC, and SOX9 expression (Fig. 5a,b). In addition, the gap-junction inhibitor carbenoxolone also suppressed ES-driven condensation and ES-driven increases of expression of the chondrogenic markers (Fig. 5a,b). This result indicates that ES induces chondrogenesis by activating the release of paracrine factors and the gap-junction activity.

### TGF-β signaling mediates MSC condensation and chondrogenesis following ES

**Figure 6:**
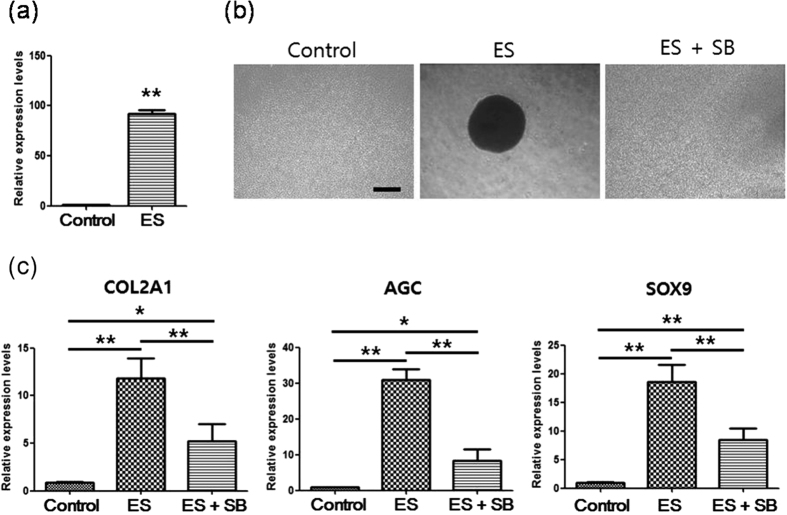
TGF-β signaling mediates MSC condensation and chondrogenesis following ES. (**a**) Real-time gene expression analysis of TGF-β1 in micromass cultures of MSCs after culture for 3 days in maintenance medium (control) or with ES (ES); Data are presented as means ± S.D. and differences were identified using Students t-test, **p < 0.01 (**b**) Effects of SB-431542 (SB) on ES-driven MSC condensation; MSCs in micromass culture were examined using phase contrast images after 3 days culture in maintenance medium (control), with ES (ES), or with ES plus SB-431542 (ES + SB); Scale bars, 500 μm. (**c**) Effects of SB on ES-induced enhancement of type II collagen (COL2A1), aggrecan (AGC), and Sox9 expression; After 3 days culture in micromass cultures without treatment (control), with ES (ES), or with ES plus SB-431542 (ES + SB), gene expression was analyzed in MSCs using real-time PCR. Data are presented as means ± S.D. Differences were identified using ANOVA; **p < 0.01, *p < 0.05.

TGF-β signaling reportedly induces prechondrogenic condensation and chondrogenesis through ATP oscillations^39^. The present study showed that ES led to much higher mRNA expression of TGF-β1 (74 fold) than expression in control cells (Fig. 6a), suggesting that TGF-β signaling is involved in ES-driven MSC condensation and chondrogenesis. In agreement, inhibition of TGF-β signaling by SB-431542 almost completely blocked ES-driven condensation and significantly suppressed ES-driven increases of COL2A1, AGC, and SOX9 expression (Fig. 6b,c). Although these data indicate that ES induces MSC condensation and chondrogenesis by activating TGF-β signaling, SB-431542 did not completely suppress ES-driven induction of chondrogenic markers (Fig. 6c), suggesting that other growth factors and cytokines also mediate the actions of ES.

### BMP signaling mediates ES-induced chondrogenesis, but not ES-induced condensation

**Figure 7:**
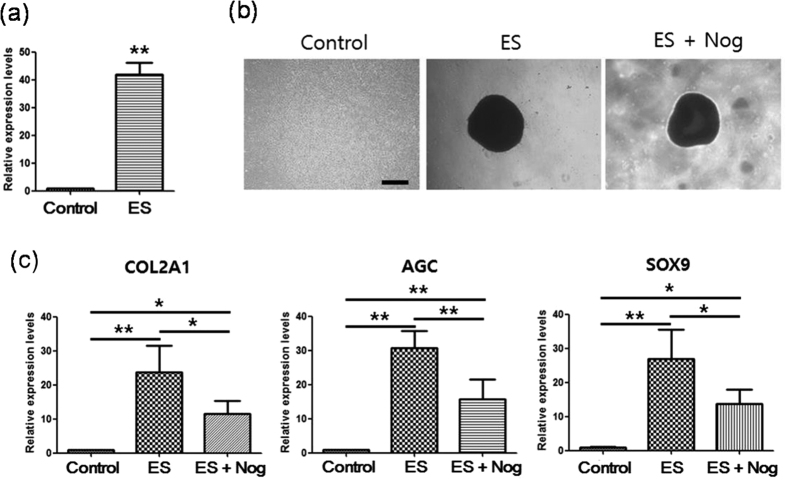
BMP signaling mediates ES-driven chondrogenesis, but not ES-driven condensation. (**a**) Real-time gene expression analysis of BMP2 in micromass cultures of MSCs after 3 days in maintenance medium (control) or in maintenance medium with ES (ES). Data are presented as means ± S.D. and differences were identified using Students t-test, **p < 0.01 (**b**) Effect of noggin (Nog) on ES-induced MSC condensation; MSCs in micromass culture were examined using phase contrast images after 3 days culture in maintenance medium (control), with ES, or with Nog and ES; Scale bars, 500 μm. (**c**) Effect of Nog on ES-induced enhancement of type II collagen (COL2A1), aggrecan (AGC), and Sox9 expression. After 3 days micromass culture of MSCs with no treatment (control), with ES (ES), or with ES and Nog, gene expression was analyzed using real-time PCR. Data are presented as means ± S.D. Differences were identified using ANOVA; **p < 0.01, *p < 0.05.

BMPs have been shown to play important roles in cartilage development^54^,^55^. Moreover, the present experiments showed that in comparison with non-treated controls, ES increased BMP2 expression by 42 fold (Fig. 7a). In addition, the inhibitor of BMP signaling noggin suppressed ES-driven increases in COL2A1, AGC, and SOX9 mRNA expression (Fig. 7c). However, noggin did not suppress ES-driven condensation (Fig. 7b), indicating that BMP signaling mediates ES-driven chondrogenesis, but not ES-driven condensation.

## Discussion

ES is a versatile treatment that remains poorly understood in the context of stem cell-based therapy. Herein, we demonstrate that ES significantly enhances the expression of chondrogenic markers (Figs 2a and 3a,b), but significantly decreases COL1 expression in MSCs (Fig. 2b). These data indicate that ES induces MSC differentiation into hyaline chondrogenic cells, and provide evidence of the potential of electrically stimulated MSCs to efficiently regenerate hyaline cartilage in the absence of additional exogenous chemical factors.

Our previous results demonstrated that ATP oscillations driven by chondrogenic growth factors such as TGF beta and insulin play essential roles for prechondrogenic condensation that is the initial step of chondrogenesis by inducing oscillatory expression of proteins involved in actin dynamics, cell migration, and adhesion which leads to collective migration and adhesion^38^,^56^. The present results demonstrate that ES generates Ca^2+^/ATP oscillations in MSCs even in the absence of exogenous growth factors (Fig. 1b). Since ES directly regulates voltage-gated Ca^2+^ channels^57^, ES can drive Ca^2+^ oscillations by modulating voltage-gated Ca^2+^ channels. In addition, since extracellular ATP signaling modulates Ca^2+^ flux by producing diacylglycerol and inositol 1,4,5-triphosphate, activating protein kinase C, and by mobilizing intracellular Ca^2+^ in multiple cell types^58^, ES can induces Ca^2+^ oscillation by extracellular ATP signaling via the P2X_4_ receptor, which is supported by the present result that P2X_4_ ATP signaling mediates the actions of ES (Fig. 4). Increased Ca^2+^ levels activate ATP-consuming processes such as ion pumping and exocytosis^59^, decrease glucose consumption by inhibiting glycolytic enzymes^60^, and decrease mitochondrial ATP production by abolishing mitochondrial membrane potential^61^, indicating the negative effects of Ca^2+^ on ATP levels. Accordingly, Ca^2+^ oscillations can drive ATP oscillations. In addition, previous studies demonstrated that pulsed electrical fields or pulsed electromagnetic fields modulate cAMP levels by activating adenosine receptors such as A_2A_, A_2b_, and A_3_ receptors, which leads to activation of anti-inflammatory pathways and cellular proliferation in cartilage^62^,^63^,^64^,^65^. Our previous results showed that ATP oscillations are dependent on cAMP dynamics^38^,^40^. These results suggest that ES drive ATP oscillations by modulating cAMP levels.

We demonstrated that pharmacological inhibition of P2X_4_-mediated ATP oscillations suppressed ES-driven condensation (Fig. 4b,c). Previous study showed that Ca^2+^/ATP oscillations induced synchronized secretion of adhesion molecules and prechondrogenic condensation^38^. In agreement, extracellular ATP signaling reportedly mediates chemotaxis and morphological changes from spread to spherical shapes, and Ca^2+^ oscillations play critical roles in cell-cell communications that lead to platelet aggregation^66^,^67^,^68^. Hence, ES may induce synchronized secretion of adhesion molecules and paracrine signaling, cell migration, and spherical morphogenesis by activating extracellular ATP signaling and Ca^2+^/ATP oscillations, leading to prechondrogenic condensation.

In the present study, ES induced chondrogenesis by stimulating both TGF-β and BMP signaling (Figs 6 and 7)^69^,^70^,^71^. It was known that TGF-β signaling reportedly stimulated prechondrogenic condensation by inducing the production of fibronectin and N-cadherin, and subsequently enhanced the expression of chondrogenic markers in various *in vitro* models^69^,^70^, and BMPs also promote chondrogenesis and regulate formation of cartilage elements in the limb^71^. Moreover, BMP signaling was shown to enhances TGF-β-induced chondrogenesis^72^. In addition, ES activates voltage-sensitive sodium and calcium ion channels to induce Ca^2+^ influx^57^. Hence, because Ca^2+^ influx activates exocytotic secretion^73^, increased Ca^2+^ influx following ES may enhance secretion of TGF-βs and BMPs, likely contributing significantly to the induction of MSC chondrogenesis. These facts can explain why ES led to stronger and more rapid induction of chondrogenesis than CM supplemented with TGF-β1 (Fig. 2a).

Many studies have shown that TGF-β signaling precedes BMP signaling and effectively initiates MSC condensation, leading to increases in the size and numbers of MSC aggregates, while BMP signaling is more effective in aggregated MSCs than in low density MSCs and increases sizes but not numbers of MSC aggregates^69^,^70^,^71^,^74^. We also previously demonstrated that TGF-β signaling but not BMP signaling drives ATP oscillations, leading to prechondrogenic condensation^39^. These data suggest differential effects of TGF-β and BMP signaling pathways on chondrogenesis. Consistent with these results, the present result showed that pharmacological inhibition of TGF-β signaling suppressed ES-driven condensation (Fig. 6b), whereas inhibition of BMP signaling did not (Fig. 7b), indicating that ES-driven condensation is mediated by TGF-β signaling, but is not mediated by BMP signaling. TGF-β signaling has been shown to enhance extracellular ATP levels and thus activate extracellular ATP signaling^75^. Accordingly, TGF-β signaling is stimulated by ES and then activates P2X_4_ signaling to consequently induce MSC condensation, which suggests that P2X_4_ signaling mediates the differential effects between TGF-β and BMP signaling on chondrogenesis.

**Figure 8:**
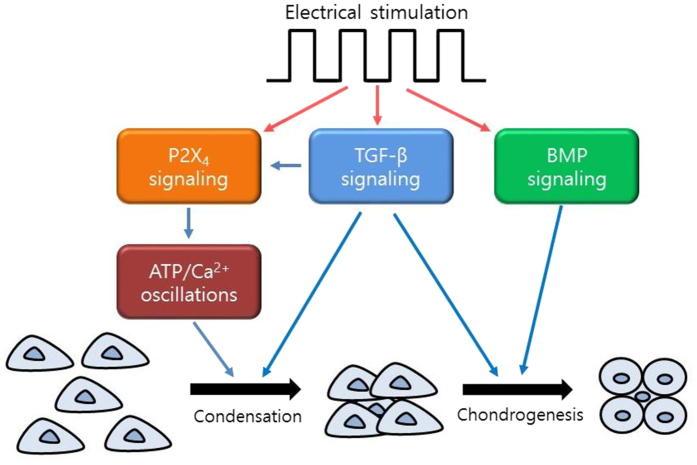
Proposed model of the functions of electrotransduction for MSC chondrogenesis. ES drives ATP/Ca^2+^ oscillations, leading to MSC condensation through TGF-β signaling and P2X_4_ signaling, and subsequently induces chondrogenesis through TGF-β signaling, BMP signaling and P2X_4_ signaling.

Based upon the findings from previous studies and the present study, the actions of ES for MSC chondrogenesis could be proposed: ES drives ATP/Ca^2+^ oscillations, leading to MSC condensation through TGF-β signaling and P2X_4_ signaling, and subsequently induces chondrogenesis through TGF-β signaling, BMP signaling and P2X_4_ signaling (Fig. 8).

In summary, in this paper we demonstrate for the first time that ES drives Ca^2+^/ATP oscillations, leading to MSC chondrogenesis in the absence of exogenous cytokine or growth factor supplements, and optimized ES regimes for induction of MSC chondrogenesis. Subsequently, we showed that P2X_4_ signaling mediates ES-driven ATP oscillations and chondrogenesis, and TGF-β and BMP signaling both mediates ES-driven chondrogenesis but have differential effects on ES-driven condensation. These data will facilitate the development of a novel ES-based technology for cell therapy and ES-based rehabilitation for cartilage repair. However, further studies are required to establish ES-based therapeutic strategies with the potential to overcome limitations of cartilage repair.

## Methods

### Cell culture and light microscopy observations

Mouse MSCs which were produced from bone marrow that was isolated from C57BL/6 mice were purchased from Invitrogen (Carlsbad, CA, USA), and were expanded in Dulbecco’s Modified Eagle’s Medium/Ham’s Nutrient Mixture F-12 (DMEM/F12; Sigma-Aldrich, St. Louis, MO, USA) with GlutaMAX-I supplemented with 10% fetal bovine serum (FBS; Invitrogen). All experiments were performed in micromass cultures. Briefly, the expanded MSCs (passage 3–7) were harvested and resuspended in maintenance medium at 2 × 10^7^ cells/ml. Droplets (10 μL) were carefully placed in each dish and cells were allowed to adhere at 37 °C for 1 h. Subsequently, 3 mL of maintenance medium were added to control and ES groups, while 3 mL of chondrogenic medium (CM; DMEM/F12, 1% ITS (Sigma-Aldrich), 10-ng/ml TGF-β1 (Peprotech, Rocky Hill, NJ, USA), 0.9-mM sodium pyruvate (Sigma-Aldrich), 50-μg/ml l-ascorbic acid-2-phosphate (Sigma-Aldrich), 10^−7^-M dexamethasone (Sigma–Aldrich), and 40-μg/ml l-proline (Sigma-Aldrich)) were added to CM group. To investigate the effects of chemical compounds on MSCs, culture medium was replaced with medium supplemented with 100-unit/ml apyrase (Sigma-Aldrich), which catalyzes the hydrolysis of ATP to AMP and inorganic phosphate, 100-μM 5-(3-bromophenyl)-1,3-dihydro-2Hbenzofuro[3,2-e]-1,4-diazepin-2-one (5-BDBD; Tocris Bioscience, Bristol, United Kingdom), which is an inhibitor of P2X_4_ purinergic receptors, 100 ng/ml noggin (R&D Systems), which is a BMP-specific antagonist protein, 10-μM SB-431542 (Sigma–Aldrich), which is an inhibitor of TGF-beta type I receptor, 100ng/ml brefeldin A (BFA), which is a inhibitor for protein secretion, and 100-μM carbenoxolone, which is a gap junction inhibitor. After 3, 7, and 14 days culture, microscope observations were performed using a phase contrast microscope (Nikon, Tokyo, Japan).

### Flow cytometry

The cell surface markers of MSCs were analyzed using a FACS Calibur flow cytometer (BD Biosciences, San Jose, CA, USA). Briefly, cells that reached 90% confluence were harvested using 0.25% EDTA and washed twice in Dulbecco’s phosphate buffered saline supplemented with 10% FBS. The cells for detecting CD11b, CD34, CD45, Sca-1, CD44 and CD73 were labeled directly with BB515 or PE-conjugated CD markers (rat anti-mouse CD11b [1: 100, BD Pharmingen; BD Biosciences, Franklin Lake, NJ, USA], rat anti-mouse CD34 [1: 100, BD Pharmingen], rat anti-mouse CD45 [1: 100, BD Pharmingen], rat anti-mouse Sca-1 [1: 100, BD Pharmingen], rat anti-mouse CD44 [1: 100, BD Pharmingen], rat anti-mouse CD73 [1: 100, BD Pharmingen]).

### Electric stimulation

ES was applied to MSCs using a C-Pace EP culture pacer (IonOptix, MA, USA), which is a multi-channel stimulator designed for chronic stimulation of bulk quantities of cells in culture. This instrument emits bipolar pulses to culture media immersed carbon electrodes of a C-dish. ES was applied to MSCs cultured under conditions of high-density micromass (2 × 10^7^ cells/ml) under electrical fields of 0, 1, 5, or 25 V/cm, with a duration of 8 ms and a frequency of 5.0 Hz. At indicated time points, MSCs were harvested in Trizol (Invitrogen) for real-time PCR analyses or were fixed using paraformaldehyde in phosphate-buffered saline (pH 7.4) for immunocytochemical analyses and alcian blue staining.

### Transfection of cells with reporter genes and bioluminescence monitoring

For real-time monitoring of intracellular ATP levels in MSCs, MSCs were transfected with a bioluminescent luciferase reporter gene (Luc) fused to an ACTIN promoter (P_ACTIN_-Luc) using Lipofectamine LTX (Invitrogen) and then the medium was replaced with recording medium (DMEM/F12 containing 10% FBS, 0.1-mM luciferin (Wako, Osaka, Japan), and 50-mM 4-(2-hydroxyethyl)-1-piperazineethanesulfonic acid (HEPES)-NaOH (pH = 7.0)). For real-time monitoring of intracellular Ca^2+^ levels in MSCs, MSCs were transfected with a aequorin gene (AQ) fused to an CMV promoter (P_CMV_-AQ) using Lipofectamine LTX (Invitrogen) and then the medium was replaced with recording medium (DMEM/F12 containing 10% FBS, 5-μM coelenterazine (Invitrogen), and 50-mM 4-(2-hydroxyethyl)-1-piperazineethanesulfonic acid (HEPES)-NaOH (pH = 7.0)). Bioluminescence intensity was continuously measured using a dish-type luminescence detector (Kronos; ATTO, Osaka, Japan) at 1 min intervals under ES.

### Lactate Dehydrogenase (LDH) Release Assays

LDH release assays were performed to assess the cytotoxicity of ES using LDH-cytotoxicity assay kits (DoGen, Korea) according to the manufacturer’s instructions. After ES for 3 or 7 days, supernatants from each dish were transferred to fresh, flat bottom 96-well culture plates containing 100-μL reaction mixtures, and were incubated for 30 min at room temperature. Formazan absorbance was then measured at 480 nm using a microplate reader (TECAN, Switzerland).

### Real-time PCR analysis

Total RNA was isolated from various MSCs cultures using the Direct-zol™ RNA MiniPrep (Zymo Research Corporation, Irvine, CA, U.S.A.) according to the manufacturer’s protocol. RNA concentrations were determined using a NanoDrop spectrophotometer (NanoDrop Technologies, Wilmington, DE, USA), and reverse transcription reactions were performed using 0.2 μg of total RNA with a TOPscriptTM cDNA synthesis kit (enzynomics, Daejeon, Korea). The real-time PCRs for beta-actin, collagen II, and aggrecan were performed using the TOPrealTM qPCR 2X Pre MIX (enzynomics). Primer sequences are listed in Table 1. Real-time PCRs were performed using a StepOnePlus™ instrument (Applied Biosystems, Grand Island, NY, USA) at 95 °C for 15 min followed by 40 cycles of denaturation at 95 °C for 10 s, extension at 60 °C for 15 s, and annealing at 72 °C for 15 s. Gene expression levels were normalized to that of beta-actin and relative gene expression was calculated using the ddCT method.

### Immunofluorescence staining and alcian blue staining

MSCs were fixed in 4% paraformaldehyde for 20 min at room temperature and were washed three times in phosphate buffered saline (PBS). Some samples were dehydrated through a graded ethanol series, infiltrated with xylene, embedded in paraffin, and sectioned at a thickness of 7-μm. After blocking in PBS containing 5% goat serum and 0.3% Triton X-100 for 60 min at room temperature, cells were incubated with rabbit anti-type II collagen antibody (1:500; EnoGene Biotech, New York, NY, USA) at 4 °C overnight, were washed three times in PBS containing 0.1% Triton X-100, and were then incubated with Alexa488-conjugated secondary antibody (1:200; Invitrogen) for 60 min at room temperature in the dark. Subsequently, cells were washed three times in PBS containing 0.1% Triton X-100 and nuclei were stained with Hoechst 33258 (Dojindo, Tokyo, Japan). To visualize accumulation of sulfated glycosaminoglycans (GAGs), cells were rinsed with PBS, fixed in paraformaldehyde for 20 min, stained with Alcian Blue Solution (pH 2.5; Nacalai tesque, INC, Japan) overnight at room temperature, and were then rinsed with distilled water three times. Accumulations of glycosaminoglycans were captured using a digital camera (Olympus, Tokyo, Japan). Expression levels of type II collagen and GAGs were quantified using immunofluorescence and alcian blue intensity profiles with the NIH IMAGE J program, and data were transferred into Microsoft Excel for further analyses.

### Statistical analysis

The results are presented as means ± SD for all samples. The statistical differences between groups were analyzed by Students t-test, and multiple comparisons were performed by Fisher’s protected least significant difference (PLSD) or Dunnett’s test. A value of p < 0.05 was considered to indicate statistical significance.

## Additional Information

**How to cite this article**: Kwon, H. J. *et al*. Electrical stimulation drives chondrogenesis of mesenchymal stem cells in the absence of exogenous growth factors. *Sci. Rep.* **6**, 39302; doi: 10.1038/srep39302 (2016).

**Publisher's note:** Springer Nature remains neutral with regard to jurisdictional claims in published maps and institutional affiliations.

## Supplementary Material

Supplementary Data

## Figures and Tables

**Table 1:** The primer sequences for Real-time PCR analysis.

Gene	Forward primers	Reverse primers	Accession No.
COL2A1	AGGGCAACAGCAGGTTCACATAC	TGTCCACACCAAATTCCTGTTCA	NM031163
Aggrecan	AGTGGATCGGTCTGAATGACAGG	AGAAGTTGTCAGGCTGGTTTGGA	NM007424
SOX9	GAGGCCACGGAACAGACTCA	CTTCAGATCAACTTTGCCAGCTT	NM011448
P2X_4_	AGACGGACCAGTGATGCCTAAC	TGGAGTGGAGACCGAGTGAGA	NM011026
TGF-β1	GCTTCAGACAGAAACTCACT	GAACACTACTACATGCCATTAT	BC013738
BMP2	ACTTTTCTCGTTTGTGGAGC	GAACCCAGGTGTCTCCAAGA	NM007553
ALP	CCAACTCTTTTGTGCCAGAGA	GGCTACATTGGTGTTGAGCTTT	NM007431
COL1	GCTCCTCTTAGGGGCCACT	CCACGTCTCACCATTGGG	NM007742
aP2	GTGTGATGCCTTTGTGGGAAC	CCTGTCGTCTGCGGTGATT	NM024406
β-actin	AGGTCATCACTATTGGCAACGA	ATGGATGCCACAGGATTCCA	NM007393
