# Supplementary material for: Electrical stimulation drives chondrogenesis of mesenchymal stem cells in the absence of exogenous growth factors
Source: Sci Rep. 2016 Dec 22;6:39302. doi: 10.1038/srep39302 (PMC5177962; doi:10.1038/srep39302)
Supplement: Supplementary Data [file srep39302-s1.pdf]

Electrical stimulation drives chondrogenesis of mesenchymal stem cells in the absence of exogenous growth factors

Hyuck Joon Kwon[1*], Gyu Seok Lee[2], and Honggu Chun[3*]

[1]Department of Physical Therapy and Rehabilitation, College of Health Science, Eulji University, Gyeonggi, Korea

[2]Department of Microbiology and Molecular Biology, College of Bioscience and Biotechnology, Chungnam National University, Daejeon, Republic of Korea

[3]Department of Bio-convergence Engineering, Korea University, Seoul, Korea,

[*]kwonhj@eulji.ac.kr, [*]chunhonggu@korea.ac.kr

SUPPLEMENTARY FIG.S1.

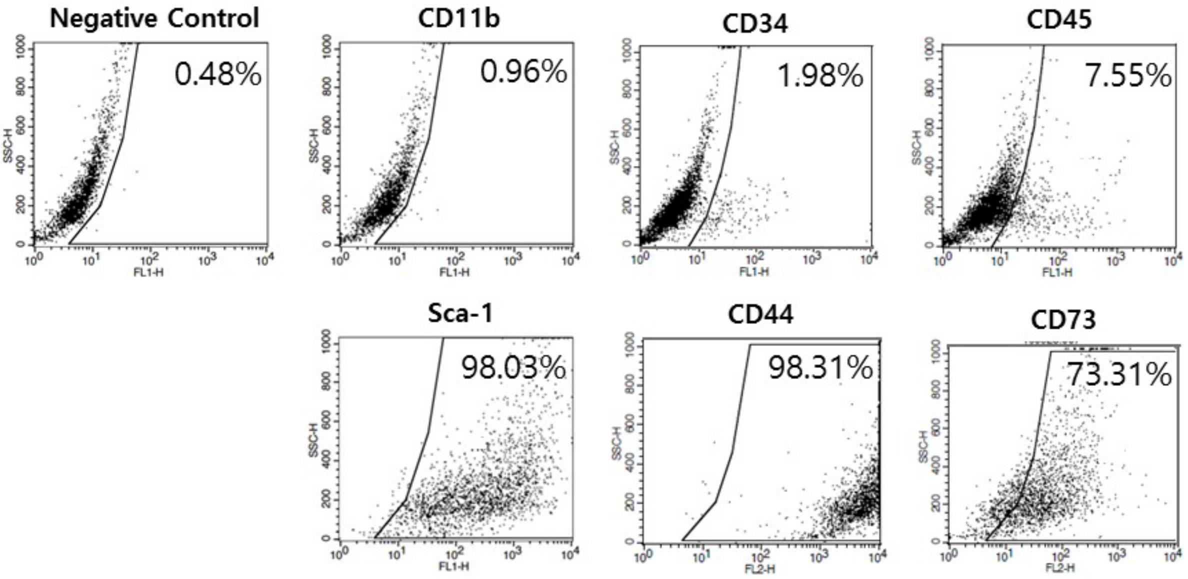

FIG. S1. Representative FACS dot plots showing the expression of CD11b, CD34, CD45, Sca-1, CD44 and CD73 in MSCs.

SUPPLEMENTARY FIG.S2.

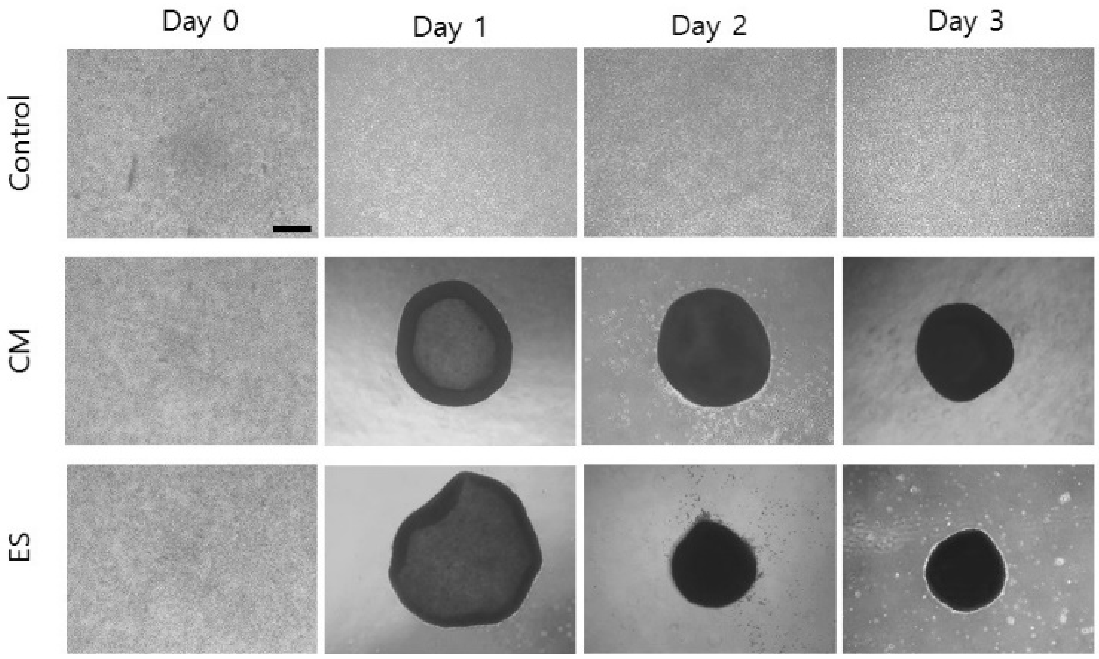

FIG. S2. Time-course observations of condensation behaviours of MSCs in micromass culture were examined with phase contrast images during culture for 3 days in maintenance medium (control), chondrogenic medium (CM) and maintenance medium under ES (ES), respectively. Scale bars, 500 μm.

SUPPLEMENTARY FIG.S3.

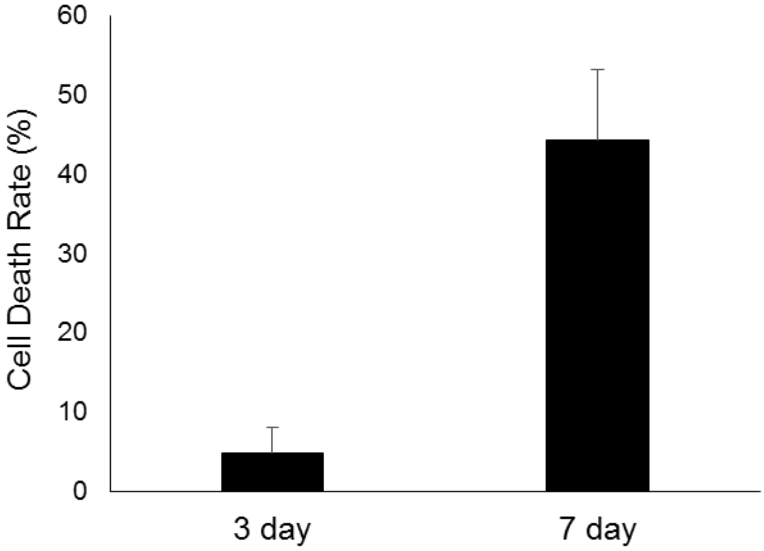

FIG. S3. Lactate dehydrogenase (LDH) leakage assay was performed for assessing the cytotoxicity of ES after 3 and 7 days under ES, respectively. Bars represent the mean ± SD of measurements from four micromass culture sets.
